# Discovery of the rosalexin pathway expands the modular network of maize diterpenoid phytoalexins

**DOI:** 10.21203/rs.3.rs-10657938/v1

**Published:** 2026-09-16

**Authors:** Anna E. Cowie, Gabrielle Wyatt, Siena Schumaker, Ahmed Khalil, Alicia Ross, Shamita Bhattacharjee, Jishnu Narayanan S. J., Yezhang Ding, Elly Poretsky, David Hurd, Jedidiah O. Peek, Alisa Huffaker, Dean J. Tantillo, Dan T. Major, Eric A. Schmelz, Philipp Zerbe

**Affiliations:** 1 Department of Plant Biology, University of California-Davis, Davis, CA 95616, USA.; 2 Section of Cell and Developmental Biology, University of California-San Diego, La Jolla, CA 92093, USA.; 3 Department of Chemistry, University of California-Davis, Davis, CA 95616, USA.; 4 Department of Chemistry and Institute for Nanotechnology and Advanced Materials, Bar Ilan University, Ramat Gan 5290002, Israel.

**Keywords:** Zea mays, terpenoids, diterpene synthases, cytochrome P450 monooxygenases, phytoalexins, plant immunity, plant specialized metabolism, plant-environment interactions, Biological Sciences, Plant Biology, Biochemistry

## Abstract

The evolutionary expansion of specialized metabolism has shaped the ability of plants to adapt to combined pest, disease, and environmental pressures. In maize (*Zea mays*), the duplication and divergence of ancestral gibberellin pathway genes have given rise to specialized kauralexin and dolabralexin diterpenoids that serve core functions in stress resilience. Here we identify rosalexins as a previously unrecognized branch of the maize diterpenoid defense network and show how duplication of hormone-metabolic genes generated a new biosynthetic pathway with distinct chemical and biological functions. By integrating genomics-enabled gene discovery, combinatorial enzyme assays, metabolomics, and AI-assisted enzyme mechanistic studies we show that rosalexin biosynthesis proceeds via an unusual 5-rosanol scaffold formed from *ent*-copalyl diphosphate through the sequential activity of ZmTPS38/CPS2/AN2 and ZmTPS42/KSL1. Further oxygenation by the promiscuous P450 enzyme, ZmCYP71Z18, yields epoxyrosanol. Unexpectedly, only epoxyrosanol strongly inhibited fungal pathogen growth, whereas neither its immediate precursor nor its downstream hydrolysis product showed comparable activity, identifying epoxidation as a key determinant of antibiotic efficacy. Large variation in rosalexin abundance exists across maize genotypes due to *ZmTPS42/KSL1* gene sequence variation and pseudogenization. Although no dominant pathogen resistance phenotype associated with rosalexin abundance was observed in maize plants, transcriptomics and metabolomics studies demonstrated the pathogen-elicited accumulation of rosalexins in maize lines featuring functional *ZmTPS42/KSL1* genes. Together, these findings reveal how duplication and functional divergence of hormone-metabolic genes generated modular diterpenoid defense layers, illustrating how plants expand chemical immunity through reuse of existing metabolic scaffolds.

## Introduction

**Fig. 1 ∣:**
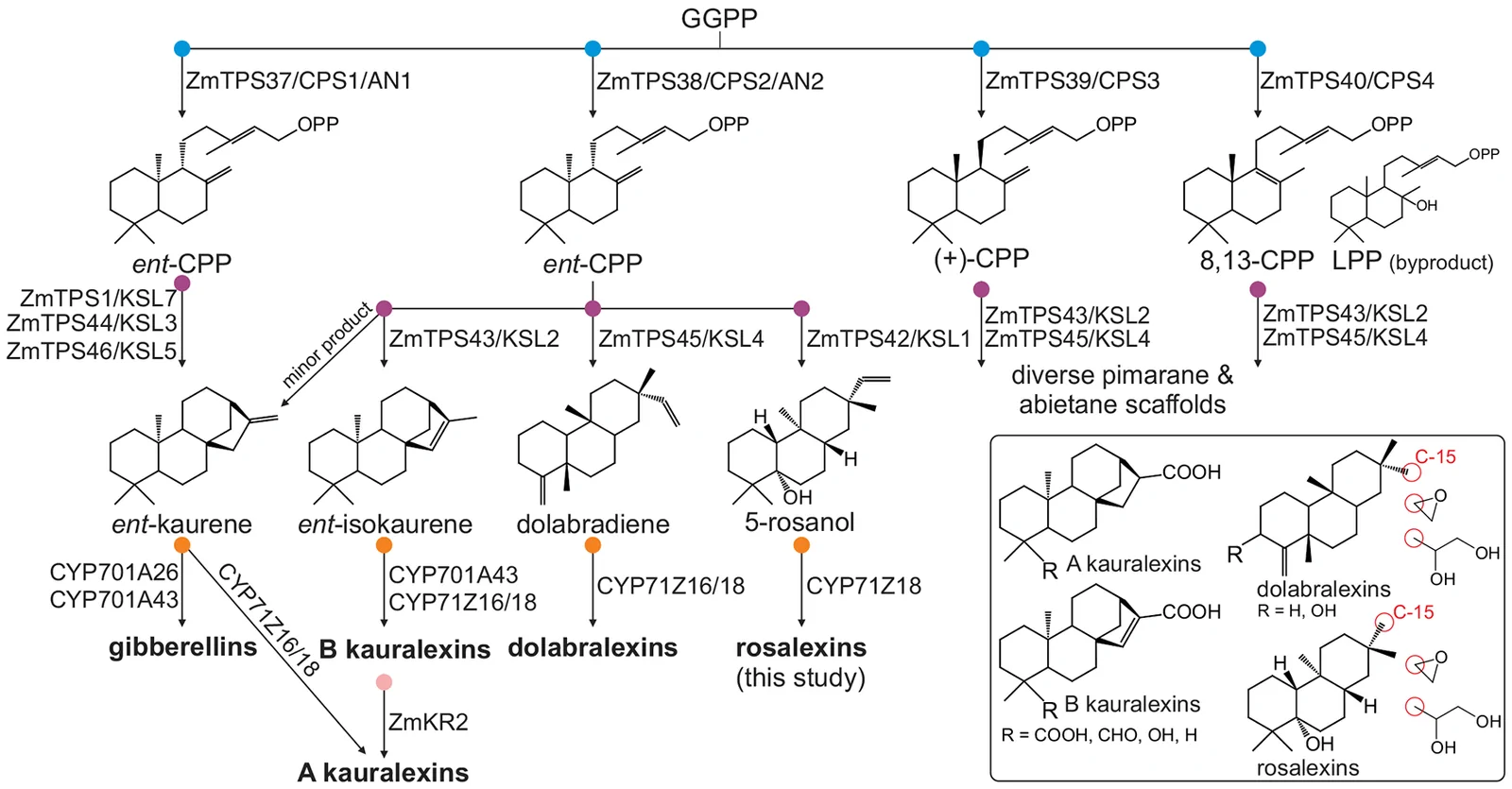
Schematic overview of the maize diterpenoid-biosynthetic network. Maize contains a modular diterpenoid-metabolic network to form specialized compound groups with distinct bioactivities, including A- and B-type kauralexins, dolabralexins, and rosalexins (this study). Abbreviations: GGPP, geranylgeranyl diphosphate; CPP; copalyl diphosphate; CPS, copalyl diphosphate synthase; KSL, kaurene synthase-like.

Plants deploy complex blends of specialized metabolites to mediate interactions with their environment, enabling their successful colonization of various ecological niches ^1,2^. Among these metabolites, the large group of diterpenoids serves multi-facetted functions with direct impact on plant vigor and fitness ^3^. Widely conserved diterpenoids such as gibberellin (GA) phytohormones play vital roles in plant development. By contrast, specialized diterpenoids are narrowly distributed across plant lineages, often display stimulus- and tissue-specific accumulation, and function in mediating the outcome of defensive and cooperative plant-environment interactions ^3,4^. However, many components of these specialized metabolite networks remain to be discovered even in well-studied poaceous grain crops. Examples include specialized diterpenoid phytoalexins that serve as core chemical defenses against microbial diseases, insect pests and abiotic stressors in maize (*Zea mays*) and rice (*Oryza sativa*) ^5,6^. Diterpenoid pathways with demonstrated or predicted functions in stress defense also exist in wheat (*Triticum aestivum*) ^7^, millet (*Setaria italica*) ^8^, barley (*Hordeum vulgare*) ^9^, and switchgrass (*Panicum virgatum*) ^10,11^, illustrating the broad distribution of species-specific networks that produce bioactive diterpenoids in monocot crops. Driven by environmental selection pressures, this expansion of diterpenoid metabolism has been enabled by the iterative duplication and functional divergence of ancestral pathway genes of general metabolism ^1,12,13^. In particular, GA-biosynthetic diterpene synthases (diTPSs) and cytochrome P450 monooxygenases (P450s) have provided genetic templates for the duplication and functional evolution of specialized enzyme functions ^12,14^. The resulting diTPS and P450 gene families comprised of functionally distinct members form modular pathway networks, where different combinations of class II and class I diTPSs and P450s generate various structural scaffolds and functional decorations that yield diterpenoids with distinct physiological functions ^3,14,15^. In maize, this modular metabolic network generates unique groups of diterpenoid phytoalexins, including kauralexins and dolabralexins, alongside bioactive zealexin, α-santalenoic acid and costic acid sesquiterpenoids ^16-22^ (Fig. 1). Kauralexins exhibit strong antimicrobial and insecticidal activities, and accumulate locally at the site of attack predominantly in above-ground tissues ^16,19,23^. Dolabralexins are largely root-specific, formed in response to both biotic and abiotic stress, and may contribute to rhizosphere microbiome interactions ^4,16-18,24^. Due to their evolutionary relationship, GAs, kauralexins and dolabralexins share a common *ent*-copalyl diphosphate (*ent*-CPP) precursor ^12,16^ (Fig. 1). Dysregulation of GA and defensive diterpenoid pathways in maize is mitigated by the presence of two duplicated class II diTPSs, ZmTPS37/CPS1/ZmAN1 and ZmTPS38/CPS2/ZmAN2, that convert the common geranylgeranyl diphosphate (GGPP) substrate into *ent*-CPP, yet display distinct spatiotemporal regulation and stress-inducibility. While ZmTPS37/CPS1/ZmAN1 functions in GA metabolism ^25^, ZmTPS38/CPS2/ZmAN2 is stress-inducible and involved in specialized diterpenoid metabolism ^23,26^. Downstream, two class I diTPSs, namely ZmTPS43/KSL2 and ZmTPS45/KSL4, control key pathway nodes by converting *ent*-CPP into the kauralexin precursor *ent*-isokaurene or the dolabralexin precursor dolabradiene, respectively ^16-18^. Catalytically promiscuous P450s of the CYP701A and CYP71Z subfamilies that are capable of functionally modifying both precursors then bring about the biosynthesis of different, bioactive kauralexins and dolabralexins ^16,17^. Presence of two additional specialized class II diTPSs, ZmTPS39/CPS3 and ZmTPS40/CPS4 that form (+)-CPP and 8,13-CPP, respectively, further expands the metabolic landscape of maize diterpenoid metabolism ^27^.

Here, we report that the maize class I diTPS, ZmTPS42/KSL1, catalyzes a previously unrecognized reaction that transforms *ent*-CPP into a 5-rosanol scaffold. Modular activity of ZmTPS38/CPS2/ZmAN2, ZmTPS42/KSL1, and the promiscuous P450 enzyme, ZmCYP71Z18, form a third *ent*-CPP-derived pathway branch in maize en route to specialized diterpenoids, coined rosalexins. *In vitro* antimicrobial activity of epoxyrosanol along with pathogen-induced *ZmTPS42/KSL1* transcript and rosalexin accumulation in aerial tissues indicated a defense-related function. However, no clear pathogen-susceptible phenotype was observed in *ZmTPS42/KSL1*-deficient maize plants, suggesting a more specialized stress-responsive function within the network of bioactive kauralexin, dolabralexin, and rosalexin diterpenoids in maize.

## Results

### ZmTPS42/KSL1 shows high gene sequence variation in maize

**Fig. 2 ∣:**
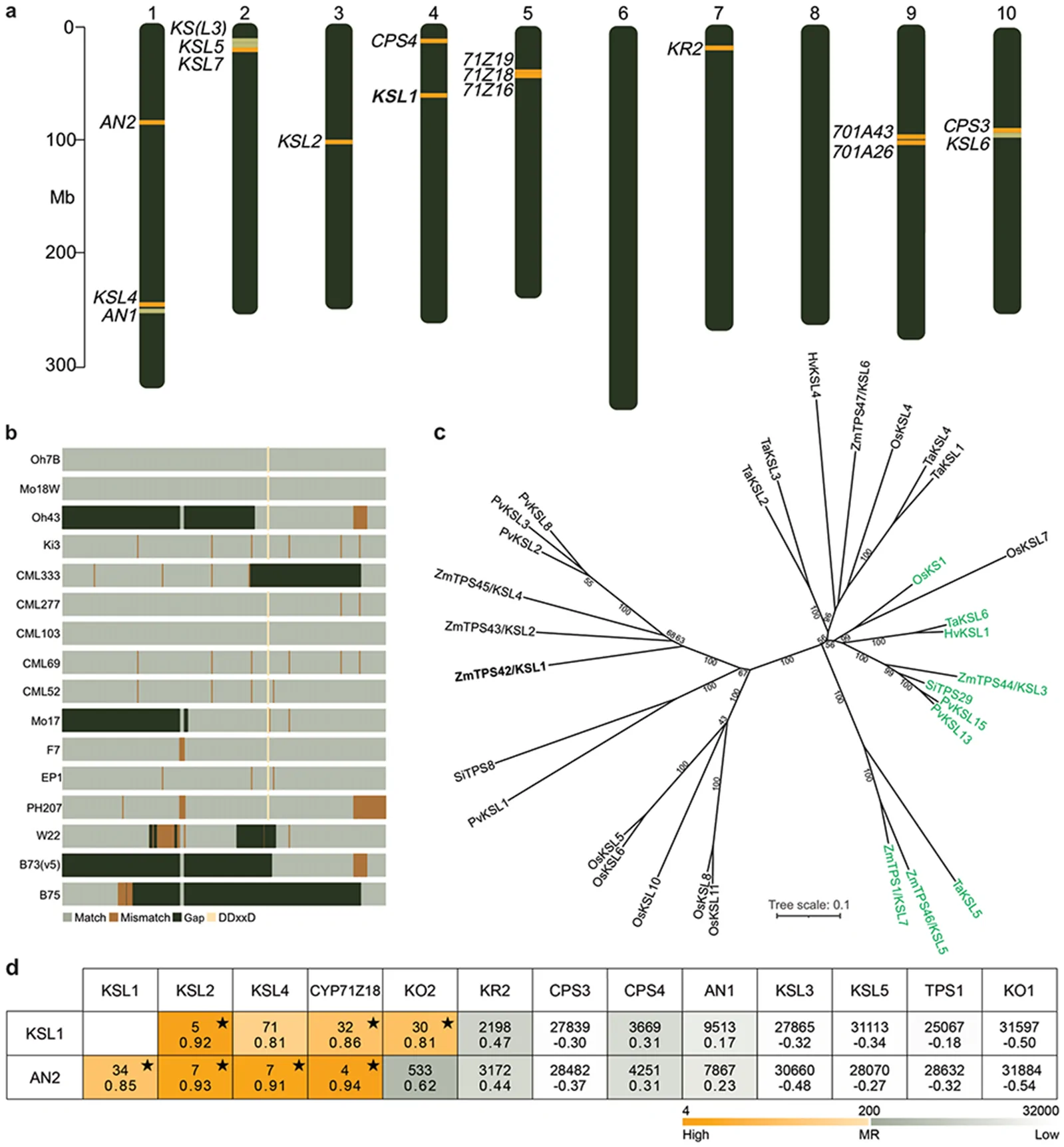
*Zm*TPS42/KSL1 is a diterpene synthase with high sequence variation across maize genotypes. **a**, Chromosome plot (Zm-B73-REFERENCE-NAM-5.0) depicting genome locations of diterpenoid-metabolic genes. Genes associated with gibberellin metabolism (green) or specialized diterpenoid metabolism (orange) are highlighted, **b**, Protein sequence alignment of ZmTPS42/KSL1 sequences derived from selected maize lines, **c**, Unrooted maximum likelihood phylogenetic tree (500 bootstrap repetitions) of ZmTPS42/KSL1 from the Oh7B line and biochemically characterized monocot class I diTPSs. Known GA-metabolic enzymes are depicted in green, **d**, Heat map illustrating the pairwise gene correlation for *ZmTPS38/CPS2/AN2* and *ZmTPS42/KSL1* genes with class II diTPS and GA metabolism genes. Strong co-expression was defined by Pearson’s correlation >0.7 and mutual rank (MR) of <200). Complementary Pearson’s correlation coefficient (PCC) of >0.7 and mutual rank value (MR) of <200 are shaded in orange (using -log10(MR)), and weak correlations in grey. PCC and MR values are printed on bottom and top respectively. Stars denote gene pairs with PCC>0.8 and MR <50. Abbreviations: CPS, copalyl diphosphate synthase; KSL, kaurene synthase-like; CYP, cytochrome P450; KO, kaurene oxidase; KR, kauralexin reductase; Ta, *Triticum aestivum;* Hv, *Hordeum vulgare;* Zm, *Zea mays;* Os, *Oryza sativa;* Pv, *Panicum virgatum;* Si, *Setaria Italica.*

Unlike related Poaceous grain crops such as rice, wheat, and barley, where gene clusters for species-specific specialized diterpenoid pathways have been identified ^7,28^, gene loci of diterpenoid pathways, including known GA, kauralexin, and dolabralexin biosynthesis are broadly dispersed across the maize genome (Fig. 2a). Mining of the maize B73 genome (Zm-B73-REFERENCE-NAM-5.0) located *ZmTPS42/KSL1* (*Zm00001eb176190*) on chromosome 4 (chr4:55755497-55756759) within a distance of 48.7 Mb to the next known terpenoid-metabolic gene, i.e., the labda-8,13-dien-15-yl diphosphate synthase, *ZmTPS40/CPS4* ^27^. However, closer inspection revealed that *ZmTPS42/KSL1* in the B73 line lacks the γ and β-domains as well as the catalytic DDxxD motif and, thus, likely represents a pseudogene (Fig. 2b). We therefore examined the *ZmTPS42/KSL1* sequences across 32 selected inbred lines, including founder lines of the Nested Association Mapping (NAM) population and the Goodman diversity panel, which revealed substantial sequence variations in the *ZmTPS42/KSL1* gene (Fig. 2b, **Supplementary Fig. S1**). Similar to B73, several lines including Mo17, CML333, B73, B75, and W22, featured major sequence variations and gaps, predictably rendering the encoded proteins non-functional. By contrast, Oh7B, EP1, CML52, CML69, and CML277 among other lines contain full-length *ZmTPS42/KSL1* genes with predicted protein sequence identities of 74-100%. Using ZmTPS42/KSL1 from the maize line Oh7B as a representative full-length protein, phylogenetic analysis placed ZmTPS42/KSL1 separate from known *ent*-kaurene synthases involved in GA metabolism and within a group of class I diTPSs that include the *ent*-iso-kaurene synthase, ZmTPS43/KSL2, the dolabradiene synthase, ZmTPS45/KSL4, as well as specialized class I diTPSs from switchgrass, thus suggesting a related function in specialized metabolism (Fig. 2c).

Gene expression profiles available in public repositories including the Sekhon gene expression atlas showed highest expression of *ZmTPS42/KSL1* in above-ground tissues including stem internodes, first and post-pollination leaves ^29,30^ as well as embryo tissue ^31^. In addition, integrating mutual rank (MR) and Pearson’s correlation (PCC) analyses of in-house transcriptome data of maize stems infected with the major maize pathogen *Fusarium verticillioides* showed patterns of co-expression of *ZmTPS42/ZmKSL1* and known genes of maize specialized diterpenoid metabolism (Fig. 2d, **Supplementary Table S1**). Specifically, *ZmTPS42/ZmKSL1* gene expression was strongly correlated with *ZmTPS43/KSL2, ZmTPS45/KSL4, ZmCYP71Z18,* and *CYP701A43/KO2* involved in kauralexin and dolabralexin metabolism. Notably, among the four maize class II diTPSs, *ZmTPS42/ZmKSL1* showed the strongest co-regulation with the *ent*-CPP synthase *ZmTPS38/CPS2/ZmAN2*. By contrast, GA-metabolic genes, including *ZmTPS37/CPS1/AN1*, *ZmTPS44/KS(L3)/D5*, and *ZmTPS46/KSL5* showed distinct expression patterns from *ZmTPS42/ZmKSL1* (Fig. 2d).

### ZmTPS42/KSL1 forms a rosane-type diterpene scaffold

**Fig. 3. ∣:**
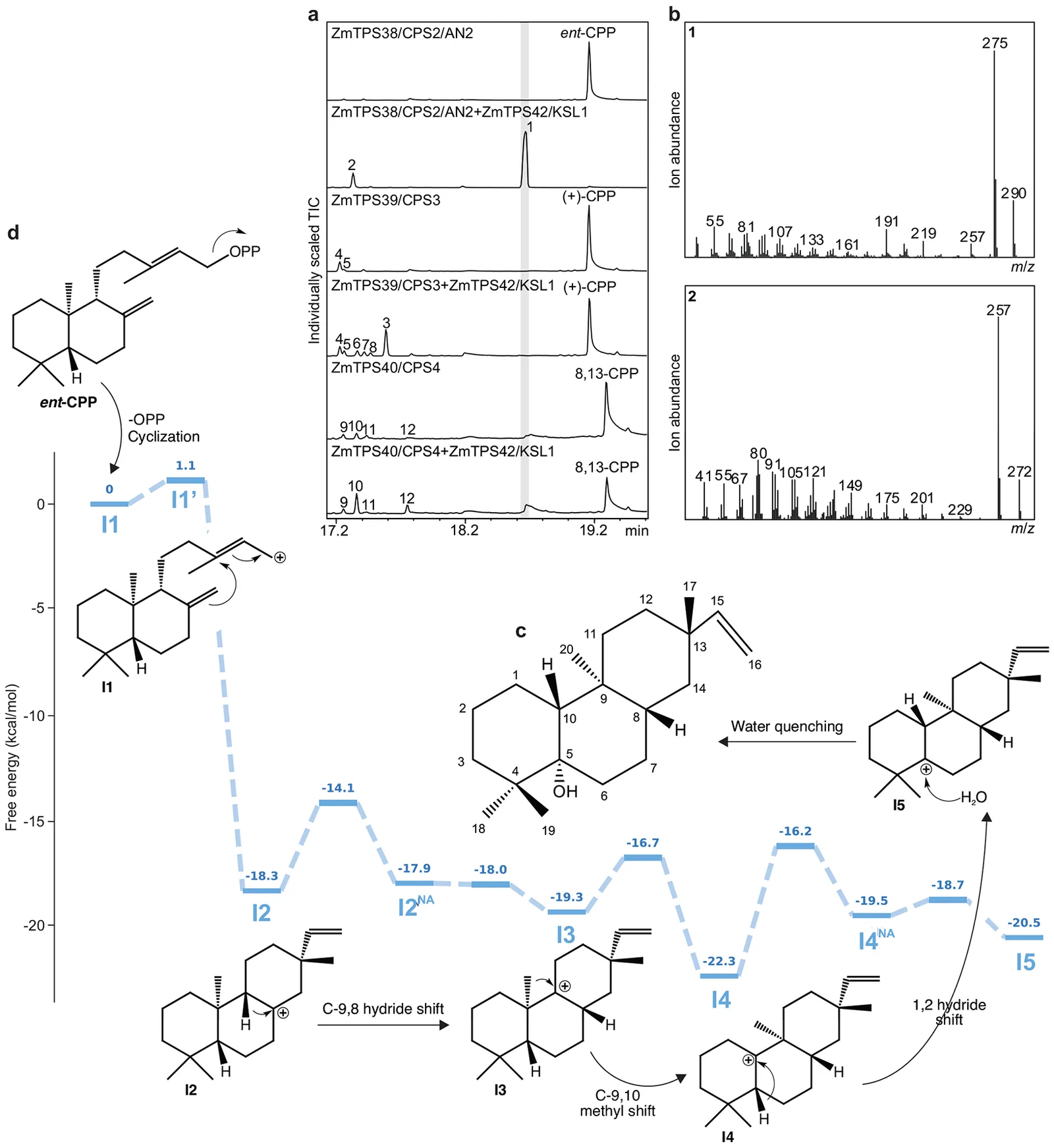
Functional characterization of *Zm*TPS42/KSL1. **a**, Total ion chromatograms (TIC) of reaction products resulting from *Agrobacterium*-mediated *Nicotiana benthamiana* co-expression assays of ZmTPS42/KSL1 with ZmTPS38CPS2/AN2, ZmTPS39/CPS3, or ZmTPS40/CPS4. **b**, Mass spectrum of compound 1 and 2 resulting from the pairwise reaction of ZmTPS38/CPS2/AN2 and ZmTPS42/KSL1. **c**, Structural identification of compound **1** as 5-rosanol, as verified by NMR analysis. **d**, Free energy diagram leading to the final intermediate at M06-2X/def2-SVP level of theory underlying the proposed ZmTPS42/KSL1-catalyzed reaction mechanism of the conversion of *ent*-CPP into 5-rosanol.

To test the biochemical function of ZmTPS42/KSL1 in the maize diterpenoid network, we selected the full-length ZmTPS42/KSL1 from Oh7B to assay pairwise enzyme activities with all maize class II diTPSs: the *ent-*CPP synthase ZmTPS38/CPS2/AN2 (catalytically identical to ZmTPS37/CPS1/AN1) ^26^, the (+)−CPP synthase ZmTPS39/CPS3, and the 8,13-CPP synthase ZmTPS40/CPS4 ^27^. *Agrobacterium*-mediated transient co-expression assays in *Nicotiana benthamiana* were conducted by pairing ZmTPS42/KSL1 individually with each class II diTPS. Co-expression of ZmTPS42/KSL1 with *Zm*TPS38/CPS2/AN2 resulted in the formation of a major product (compound **1**) and a minor product (compound **2**). GC-MS analysis of compound **1** showed *m*/*z* 275 and *m*/*z* 290 mass ions indicative of a hydroxylated labdane-type diterpene and product **2** featured *m/z* 257 and *m/z* 272 mass ions characteristic of a labdane olefin scaffold (Fig. 3a-b). In addition, the pairwise activity of ZmTPS42/KSL1 with the (+)−CPP synthase ZmTPS39/CPS3 yielded several labdane diterpenoids (compounds **3-8**), albeit at very low abundance. Among these, **3**, **6**, **7,** and **8** were distinct ZmTPS42/KSL1 products and only **3** could be identified as sandaracopimaradiene based on comparison to an authentic standard (Fig. 3a, **Supplementary Fig. S2**). Combined activity of ZmTPS42/KSL1 with the 8,13-CPP synthase ZmTPS40/CPS4 did not result in any new products beyond those also observed in the expression of ZmTPS40/CPS4 alone (unidentified compounds **9-12**). However, compounds **10** and and **12** showed higher abundance when ZmTPS40/CPS4 was co-expressed with ZmTPS42/KSL1 (Fig. 3a, **Supplementary Fig. S2**).

Given the high level of gene co-expression of *ZmTPS42/KSL1* with *ZmTPS38/CPS2/AN2* but not *ZmTPS39/CPS3* or *ZmTPS40/CPS4* (Fig. 2d), we investigated this pairwise reaction further. To elucidate the structure of the ZmTPS42/KSL1 product **1** we enzymatically produced and purified >1 mg of compound **1** for NMR analysis and identified the metabolite as 5-hydroxy-rosa-15-ene (hereafter named 5-rosanol) (Fig. 3c, **Supplementary Fig. S3**). Complementary to experimental NMR data, we employed computational NMR analysis of 16 possible isomers across the four stereocenters present in 5-rosanol to assign absolute stereochemistry. Experimental 1D (^1^H and ^13^C) and 2D (H2BC, HMBC, COSY, NOESY) NMR data assessed with complementary DP4+ calculations matched compound **1** to stereoisomer 11, identifying the ZmTPS42/KSL1 product (5*S*,8*S*,9*S*,10*R*,13*S*)-5-hydroxy-rosa-15-ene (**Supplementary Tables S2 & S3**) ^32^. Low product abundance prevented NMR analysis of the ZmTPS42/KSL1 product **2**.

Verification of the 5-rosanol structure enabled us to apply the AI-assisted automated reaction mechanism discovery platform, RxnNet ^33^ to propose a catalytic mechanism of 5-rosanol biosynthesis catalyzed by ZmTPS42/KSL1 (Fig. 3d). Following abstraction of the pyrophosphate ester of *ent*-CPP, the reaction proceeds via cyclization of **I1** (set as the reference state with a free energy of 0.0 kcal/mol for its global minimum conformer in the gas-phase) to form the pimara-15-en-8-yl^+^ carbocation (**I2**). Notably, a slight 1.1 kcal/mol free energy barrier arises from a conformational change, which is followed by a barrierless cyclization process. A subsequent ring-puckering of the global minimum conformer of **I2** (ΔG^‡^ = 4.1 kcal/mol) results in the near-attack conformer (NAC) ^34,35^ of **I2** (**I2^NA^**). The 1,2-hydride shift between **I2^NA^** and **I3** is a barrierless process, as the latter is already in a NAC state ready for the 1,2-hydride shift. The **I3** to **I4** methyl migration proceeds via a free energy barrier of 2.6 kcal/mol. The subsequent hydride shift goes via an initial conformational change to reach **I4^NAC^** with a ΔG^‡^ = 6.1 kcal/mol followed by the chemical step with a barrier of 0.8 kcal/mol. Finally, water quenching of the carbocation center of **I5** results in 5-rosanol (**Supplementary Fig. S4**). Intermediates **I2** through **I5** exhibit similar free energies and are characterized by low-barrier carbocationic transformations. Consequently, an equilibrium may exist among these species, with the final product distribution being primarily determined by the positioning of the active-site water, which is analogous to that reported for the active-site base in taxadiene synthase ^36-38^. Based on this reaction mechanism and the characteristic *m*/*z* 257 and *m*/*z* 272 mass ions, we hypothesize that product **2** of ZmTPS42/KSL1 represents a rosadiene olefin scaffold derived from neutralization of the intermediary rosa-15–en-10-yl^+^ (**I4**) or rosa-15-en-5-yl^+^ (**I5**) carbocations via deprotonation rather than water quenching ^39^.

**Fig. 4. ∣:**
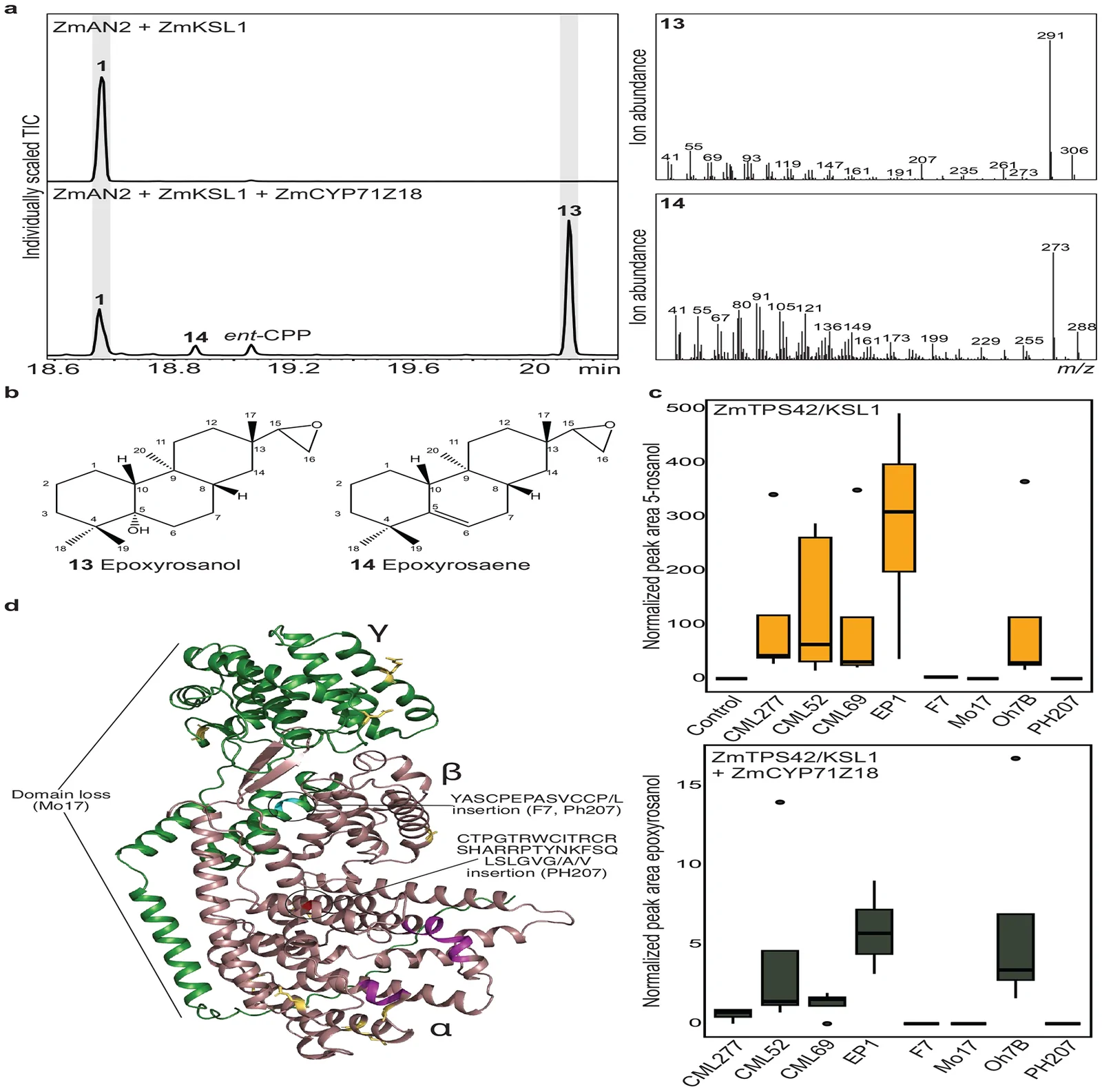
Identification of the rosalexin pathway. **a**, Total ion chromatograms (left) and mass spectra (right) of reaction products resulting from *Nicotiana benthamiana* co-expression assays of ZmTPS38/CPS2/AN2 and ZmTPS42/KSL1 only, or with the addition of ZmCYP71Z18. **b**, Structural identification via NMR analysis of compounds **13** and **14** as epoxyrosanol and epoxyrosaene, respectively. **c**, Normalized abundance of 5-rosanol (top) and epoxyrosanol (bottom) produced by ZmTPS42/KSL1 from each maize line co-expressed with *Zm*TPS38/CPS2/AN2 for 5-rosanol and ZmTPS38/CPS2/AN2 and ZmCYP71Z18 for epoxyrosanol. Error bars represent standard error of the mean (n=4). **d**, AlphaFold 3 homology model of Oh7B ZmTPS42/KSL1 (brown) with the DDxxD and NSE/DTE motifs highlighted in magenta. Sequence variations in ZmTPS42/KSL1 sequences derived from other tested lines are highlighted in yellow.

Given that the biosynthesis of related kauralexin and dolabralexin diterpenoids requires the functional modification of the respective diTPS-produced scaffolds by specialized P450 enzymes of the CYP701A and CYP71Z subfamilies ^16,17,20^, we next performed co-expression studies combining ZmTPS38/CPS2/AN2 and ZmTPS42/KSL1 (Oh7B) with known diterpenoid-metabolic maize P450s. Specifically, co-expression with ZmCYP71Z18 yielded new major (compound **13**) and minor (compound **14**) products, featuring dominant mass ions of *m*/*z* 306 and *m*/*z* 288, respectively, that are indicative of oxygenated labdane diterpenoids (Fig. 4a). Purification and NMR analysis of **13** and **14** verified these structures as 5-hydroxy-15-epoxyrosa-5(6)-ene (hereafter named epoxyrosanol) and epoxy-rosa-5(6)-ene (epoxyrosaene), respectively (Fig. 4b, **Supplementary Figs. S5 & S6**). While epoxyrosanol is derived from 5-rosanol, the formation of small quantities of epoxyrosaene supported our hypothesis that the minor ZmTPS42/KSL1 product **2** is rosaene and serves as a precursor for epoxyrosaene. In keeping with the nomenclature of other maize diterpenoids, we collectively designated 5-rosanol (**1**), rosaene (**2**), epoxyrosanol (**13**), and epoxyrosaene (**14**) as rosalexins. Contrasting ZmCYP71Z18, functional testing of ZmTPS38/CPS2/AN2 and ZmTPS42/KSL1 with other characterized maize diterpenoid-metabolic P450s, including ZmCYP71Z16 ^17^, ZmCYP71Z19 ^20^, ZmCYP701A26 ^16,40^, and ZmCYP701A43 ^16^, yielded no additional products (**Supplementary Fig. S7**).

Having established the enzymatic function of the Oh7B ZmTPS42/KSL1, we assessed the activity of predicted ZmTPS42/KSL1 proteins from other maize lines using co-expression assays with ZmTPS38/CPS2/AN2 alone or with ZmTPS38/CPS2/AN2 and ZmCYP71Z18. The partial protein sequence of Mo17 ZmTPS42/KSL1, lacking a substantial segment of the N-terminal *γβ*-domain, expectedly resulted in an inactive enzyme (Fig. 4c-d). Likewise, the full-length ZmTPS42/KSL1 proteins derived from the maize lines F7 and PH207 showed no or only trace enzyme activity, likely as a result of more extensive sequence variation and insertions (Fig. 4c-d). By contrast, rosalexin production was observed for the full-length ZmTPS42/KSL1 proteins derived from the CML277, CML52, CML69, Oh7B, and EP1 maize lines (Fig. 4c-d). Notably, 5-rosanol product levels varied significantly (p = 0.036) across ZmTPS42/KSL1 proteins from these tested maize genotypes.

### Stress-elicited rosalexin accumulation in planta reveals additional pathway products

**Fig. 5. ∣:**
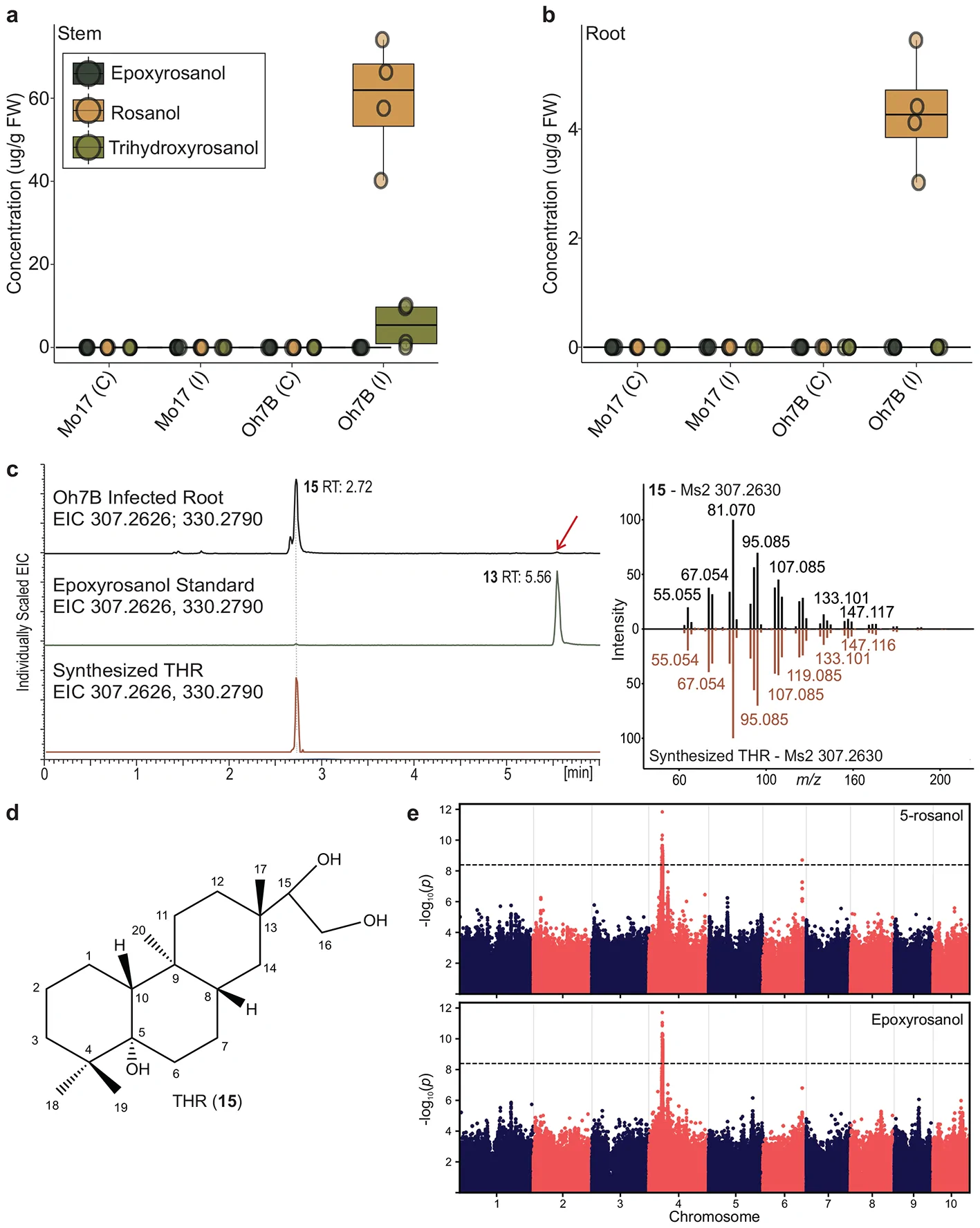
Rosalexins accumulate *in planta* in response to pathogen infection. **a-b**, LC-MS/MS average metabolite abundance in stem (**a**) and root (**b**) for epoxyrosanol, 5-rosanol and trihydroxyrosanol (THR) in Mo17 and Oh7B plants in mock-treated (control) and plants infected with *Fusarium verticillioides* (infected). Boxes represent the interquartile range (IQR), center line indicates the median, and whiskers extend to 1.5×IQR (n=4). Raw metabolite abundance data are given in **Supplementary Tables S4 & S5**. **c,** LC-MS/MS extracted ion chromatogram (EIC) at 307.2626 and 330.2790 *m/z* of infected Oh7B roots, purified epoxyrosanol standard and synthesized trihydroxyrosanol (THR) standard (left) and LC-MS2 spectra (right) comparing *in planta* compound **15** (black) and synthesized THR standard (brown). **d**, NMR verified structure of synthesized THR. **e,** Manhattan plots of the Goodman diversity panel association analysis using 5-rosanol and epoxyrosanol abundance as mapping traits. Negative log10- transformed *P* values from the compressed mixed linear model are plotted on the y axis. The dashed line denotes the 5% Bonferroni-corrected threshold for approximately 12.3 million SNP markers with the most statistically significant SNP located at position 57,031,961 (B73 RefGen_v5) on chromosome 4 for both mapping traits.

To investigate the presence of rosalexins *in planta*, we performed targeted metabolomics of maize stem and root tissues of 53-day-old plants using contrasting maize lines containing inactive (Mo17) or functional (Oh7B) *ZmTPS42/KSL1* genes three days after pathogen infection with *F. verticillioides* as compared to mock-treated (wounding only) control samples. Consistent with the absence of a functional *ZmTPS42/KSL1*, LC-MS/MS analysis via quantifying relative ion intensity and abundance showed that Mo17 tissues did not contain rosalexin metabolites at any detectable level irrespective of pathogen or mock treatment (Fig. 5; **Supplementary Tables 4 & 5**). Control Oh7B plants also did not contain detectable rosalexin levels. However, accumulation of 5-rosanol was observed in pathogen-infected stems and roots of Oh7B plants (Fig. 5a-b). Standard-verified 5-rosanol was detected by a predominant *m*/*z* 273 mass fragment representing a neutral loss of water [M-H_2_O+H]^+^. On average (n=4), infected plants accumulated 5-rosanol at 59.5 ± 14.6 μg g^−^ 1(FW) in stems and at 4.3 ± 1.1 μg g^−1^ (FW) in roots, consistent with lower *ZmTPS42/KSL1* gene expression in roots. Ion spectra analysis of epoxyrosanol revealed a *m*/*z* 307 fragment [M+H]^+^ consistent with the NMR molecular formula of the purified compound, as well as a predominant *m*/*z* 330 ion representing a sodium adduct [M+Na]^+^. Based on these ions, epoxyrosanol was detected at trace amounts (0.76 ± 0.38 ng g^−1^ FW in stems and 0.015 ± 0.015 ng g^−1^ FW in roots) of infected Oh7B plants (Fig. 5a-b). In infected Oh7B stem tissue, another abundant metabolite (compound **15**) was detected, which also featured a *m/z* of 307 mass fragment and a near identical MS2 fragmentation pattern as compared to expoxyrosanol, yet showed an earlier retention time of 2.7 min as compared to epoxyrosanol at 5.5 min (Fig. 5c-d). Our prior work had demonstrated the presence of trihydroxydolabrene (THD) as a major metabolite derived from the dolabralexin pathway in roots of field-grown maize ^17^. We therefore hypothesized that compound **15** could be a similar structure, resulting from the enzymatic or spontaneous opening of the epoxide ring of epoxyrosanol. We tested this hypothesis using a chemical conversion approach. Subjecting enzymatically produced, purified epoxyrosanol to slightly acidic conditions indeed yielded **15** (Fig. 5c-d). Further NMR analysis identified **15** as trihydroxyrosanol (THR), adding another compound to the group of rosalexins that features the loss of the epoxide and gain of two hydroxyl groups at C-15 and C-16 (Fig. 5e, **Supplementary Fig. S8**). Re-analyzing pathogen-infected plant material demonstrated the presence of THR in infected roots, albeit at trace amounts. Conversely, THR significantly accumulated in stem tissue at 5.3 μg g^−1^ (FW) (Fig 5a-b). No THR was detected in control plants.

Given the pathogen-elicited accumulation of rosalexins *in planta*, we performed additional metabolite-based Genome Wide Association Studies (mGWAS) previously used to study maize diterpenoid metabolism ^16,20^ to identify the genetic loci underlying rosalexin biosynthesis. Using the major mass ions for 5-rosanol and epoxyrosanol as the mapping traits, we detected the strongest association signals on chromosome 4. The most significant SNP, chr4_57031961, was located 1.28 Mb downstream of the genomic region containing *ZmTPS42/KSL1*, whereas the nearest SNP, chr4_55805238, was only 48 kb downstream of the gene (Fig. 5e, Supplementary Tables S6 & S7). We further re-assessed previously reported transcriptomic data ^20^, which showed that *ZmTPS42/KSL1* was the most strongly induced gene within the associated genomic region following fungal pathogen infection (**Supplementary Table S8**). By contrast, no significant association was identified in the regions on chromosomes 1 and 5 where *ZmTPS38/CPS2/AN2* and *ZmCYP71Z18* are localized, respectively.

### Rosalexins display antibiotic activity against fungal pathogens

**Fig. 6. ∣:**
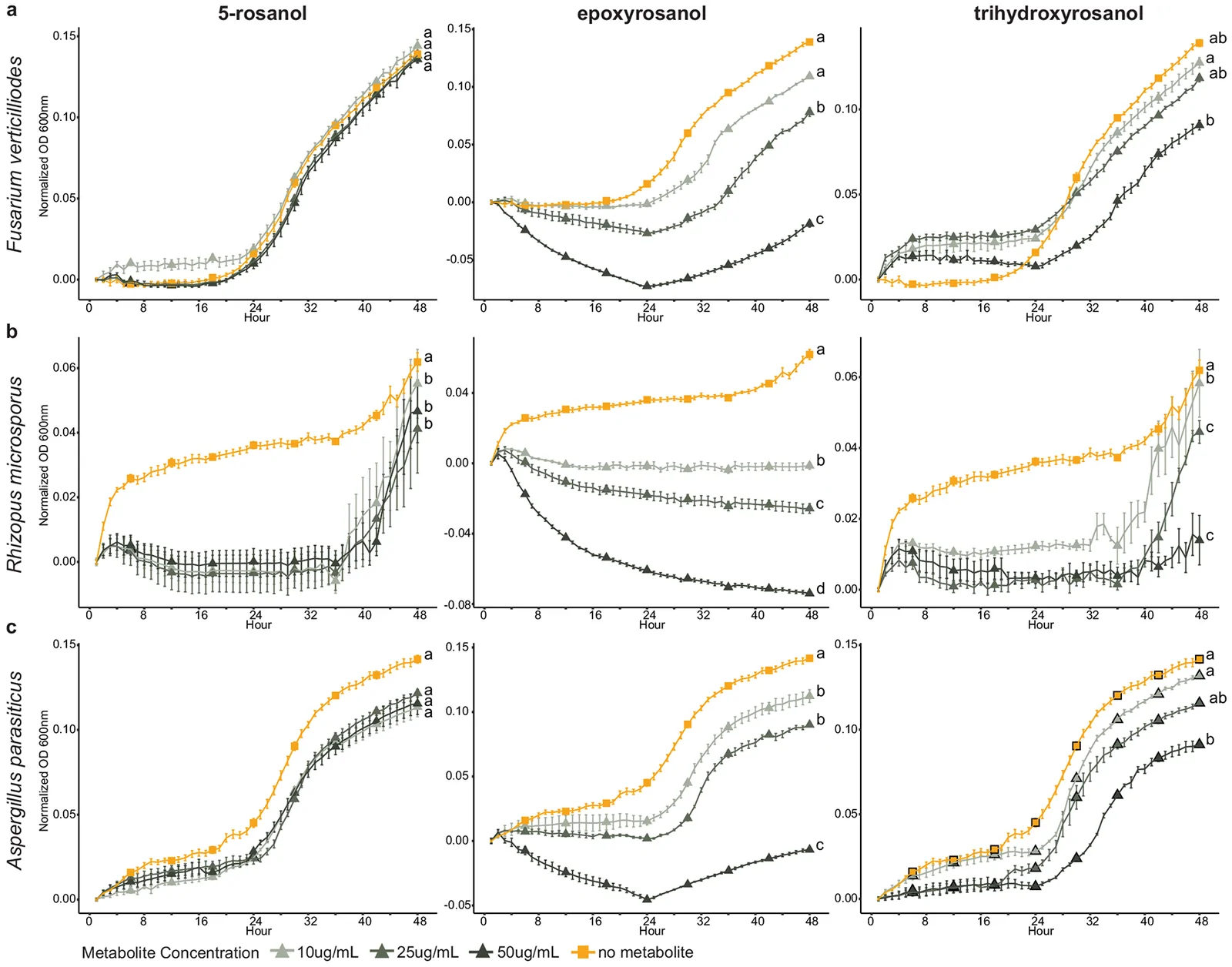
Antifungal bioactivity of rosalexins. Average growth (OD_600_) of *Fusarium verticillioides* (**a**), *Rhizopus microsporus* (**b**), *and Aspergillus parasiticus* (**c**) pathogens in liquid culture in the presence of different concentrations of purified 5-rosanol, epoxyrosanol, and trihydroxyrosanol (THR). Assays were performed over a 48-hour time course in a defined RPMI-1640 MOPS buffer solution using a microtiter plate assay. Error bars represent standard error of the mean (n=6). Different letters (a-c) represent statistically significant differences in measurements at the 48 h time point; statistical analysis was performed using an ANOVA (p < 0.05), followed by Tukey’s Honest Significance Difference (HSD) to identify significantly different treatment pairs.

To assess the antifungal potential of rosalexins, we performed *in vitro* fungal growth inhibition assays using the maize pathogens *Fusarium verticillioides*, *Aspergillus parasiticus*, and *Rhizopus microsporus*, as major causal agents of maize seedling blight, stalk, ear and root rots, and mycotoxin contamination. Fungal growth was measured by optical density at 600 nm (OD_600_) in the presence of purified 5-rosanol, epoxyrosanol, and THR. Following prior bioactivity assays of maize kauralexins and dolabralexins, we utilized concentrations of 10, 25, and 50 μg mL^−1^ for each metabolite to test for antimicrobial activity ^16,17^. With respect to *F. verticillioides*, 5-rosanol displayed no significant growth-inhibitory activity irrespective of compound concentration (Fig. 6, **Supplementary Table S9**). In the presence of THR, a brief increase in fungal growth was observed, while a moderate inhibitory effect was detected at the 48 h end point. By contrast, epoxyrosanol significantly inhibited fungal growth at all tested compound concentrations. A similar impact on fungal growth was observed for *A. parasiticus* with epoxyrosanol showing the strongest growth-inhibitory activity. On the other hand, 5-rosanol and THR had a strong inhibitory effect on the growth of *R. microsporus* for ~32 h, after which fungal growth rapidly increased to near-control levels. Similar to *F. verticillioides* and *A. parasiticus*, epoxyrosanol showed strong inhibitory activity against *R. microsporus*. For example, at a concentration of 25 μg mL^−1^, epoxyrosanol inhibited *R. microsporus* by 2.43 fold (p < 0.000000001), as compared to 1.96 fold (p = 0.000000455) for *F. verticillioides*, and 1.53 fold (p = 0.000149473) for *A. parasiticus* (Fig. 6, **Supplementary Table S9**). To assess the stability and possible metabolization of rosalexins during these fungal assays, we re-extracted the epoxyrosanol and THR from the fungal cultures at the 48 h endpoint. LC-MS/MS analysis of these extracts showed that both compounds were recovered from the fungal cultures and no new compounds or possible breakdown products were detected (**Supplementary Fig. S10**).

### Rosalexins are stress-inducible alongside other specialized maize diterpenoid pathways

**Fig. 7. ∣:**
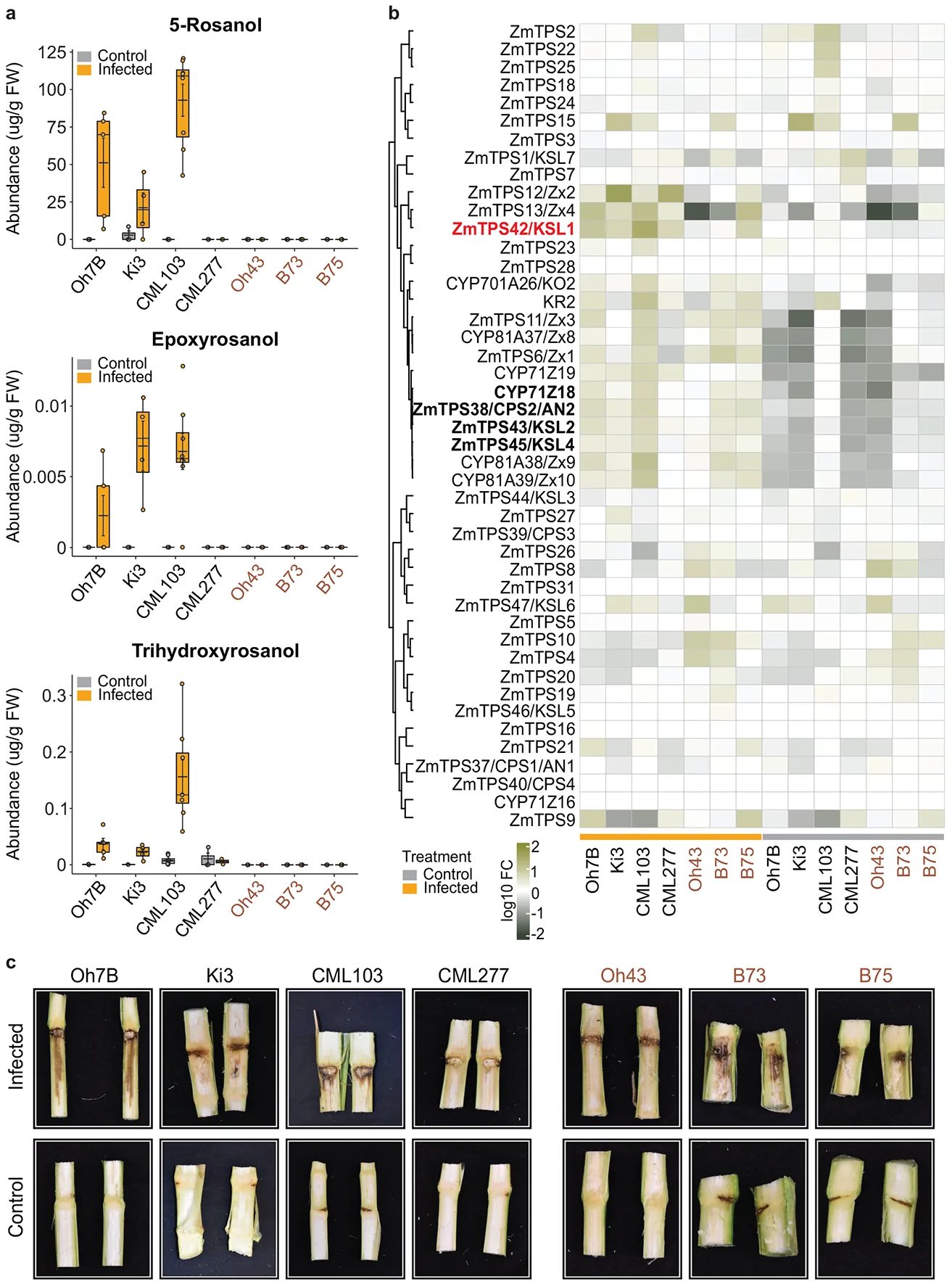
Rosalexins are pathogen-induced alongside other defense terpene pathways. **a,** LC-MS/MS average metabolite abundance in stem tissue for 5-rosanol, epoxyrosanol, and trihydroxyrosanol. **b**, heat map depicting log10 transformed fold change gene expression $\left(\log\;10(\frac{sample\;mean\;[FPKM+1]}{median\;across\;all\;samples\;(FPKM)})\right)$ across all samples (FPKM)) of known maize terpene synthases, P450 genes, and kauralexin reductase 2 (KR2) in response to infection with *F. verticillioides* or mock treated controls (n=3). **c**, Representative images of maize stems infected with *F. verticillioides* (infected) or water (control) using the lines Oh7B, Ki3, CML103, and CML277 (featuring a functional ZmTPS42/KSL1 gene) and Oh43, B73, and B75 (containing non-functional ZmTPS42/KSL1).

To investigate associations of rosalexins with pathogen responses *in planta*, available maize genotypes featuring ZmTPS42/KSL1 genes demonstrated or predicted to be functional (Oh7B, Ki3, CML103, CML277) or containing non-functional *ZmTPS42/KSL1* genes (Oh43, B73, and B75) were selected for plant pathogen stress studies (Fig. 2c). Since rosalexins are significantly more abundant in stems as compared to roots (Fig. 5), analyses focused on stem tissue. Here, longitudinal stem incisions between nodes 3 and 4 of 62-day old plants were infected with either *F. verticillioides* or water (control). One week post *F. verticillioides* infection, lines lacking a functional *ZmTPS42/KSL1* expectedly did not show abundance of rosalexins. In functional lines rosalexins were detected at varying levels in pathogen-treated plants with the exception of maize line CML277 (Fig. 7a). Parallel transcriptome analysis of stem tissue showed that *ZmTPS42/KSL1* transcript abundance was on average increased by 25.8-fold in pathogen-treated plants, with highest gene expression in CML103 consistent with high rosalexin accumulation in this line (Fig. 7b; **Supplementary Table S1**). Notably, increased *ZmTPS42/KSL1* gene expression was also observed in B75 which features a pseudogene. The remaining rosalexin pathway genes, *ZmTPS38/CPS2/ZmAN2* and *ZmCYP71Z18*, showed moderately lower transcript abundance as compared to *ZmTPS42/KSL1* with expression levels more similar to *ZmTPS43/KSL2* and *ZmTPS45/KSL4*, as the core genes controlling the committed kauralexin and dolabralexin pathway nodes, respectively. Conversely, *ZmCYP71Z16*, which appears to predominantly function in dolabralexin biosynthesis, showed low expression in stem tissue. In addition, although *ZmTPS42/KSL1* and *ZmTPS40/CPS4* co-localize albeit distantly on chromosome 4, the two genes showed significant differences in pathogen-elicited gene expression patterns with *ZmTPS40/CPS4* being more abundant in root tissue without any clear pattern of pathogen inducibility (Fig. 7b, **Supplementary Table S1**). Notably, visual inspection of control and pathogen-infected plants showed that stem lesion sizes varied across the tested maize genotypes with no distinguishable symptoms between lines featuring functional *vs* non-functional *ZmTPS42/KSL1*, respectively (Fig. 7c).

## Discussion

In light of rising environmental pressures that threaten crop health and agricultural production, understanding the dynamic networks of specialized metabolites that mediate stress defense and ecological adaptation is pivotal for improving disease control and resistance trait breeding ^4,6,41^. Species-specific blends of diterpenoid phytoalexins with potent antimicrobial, insecticidal, and allelopathic functions that directly impact plant protection and vigor exist in major grain crops, including rice, maize, wheat, and barley ^5,7,9^. This study highlights the capacity of integrated multi-omics, enzyme biochemical, computational mechanistic studies, and mGWAS approaches to uncover hidden specialized metabolic pathways even in extensively studied crops such as maize.

Prior maize research identified related yet distinct kauralexin, zealexin, and dolabralexin terpenoids that serve as core components in the protection against fungal pathogens, insect pests, and drought stress ^16-18,23-20^. These terpenoid blends arose through the functional expansion of the TPS and P450 enzyme families via repeated often transposon-mediated tandem gene duplications and gene losses that occurred during the allopolyploidization and subsequent rediploidization events that shaped the maize genome ^42,43^. As a common mechanism in terpenoid diversification, especially the iterative duplication and neo-functionalization of GA-biosynthetic genes have contributed to terpenoid chemical diversity ^12^. Indeed, such gene duplication events resulted in the evolution of two catalytically identical *ent*-CPP synthases, the GA-metabolic ZmTPS37/CPS1/AN1 and its stress-inducible paralog ZmTPS38/CPS2/AN2 ^26^. The distinct stress-responsive gene expression of *ZmTPS38/CPS2/AN2* enabled the dysregulation of GA and defensive diterpenoid metabolism in maize ^16,17^, and forms a metabolic hub for converting *ent*-CPP via different pathway modules. Here, ZmTPS43/KSL2 and ZmTPS45/KSL4 produce the committed *ent*-iso-kaurene and dolabradiene intermediates en route to kauralexins and dolabralexins, respectively ^16,17^ (Fig. 1). Phylogenetic relatedness to ZmTPS43/KSL2 and ZmTPS45/KSL4 suggests that ZmTPS42/KSL1 emerged as another gene duplication recruited from GA metabolism that functionally diverged toward specialized metabolic scaffolds. The presence of functional and pseudogenized *ZmTPS42/KSL1* genes across different maize genotypes exemplifies the high genetic diversity in maize that shapes metabolic network architecture and chemical diversity (Fig. 2). The formation of rosalexins by the combined activity of ZmTPS38/CPS2/AN2, ZmTPS42/KSL1, and ZmCYP71Z18 adds yet another pathway branch to the portfolio of *ent*-CPP-derived diterpenoids produced in maize (Fig. 1). Due to their distinct product specificity, ZmTPS42/KSL1, ZmTPS43/KSL2, and ZmTPS45/KSL4 serve as core pathway branch nodes en route to rosalexins, kauralexins, and dolabralexins. Presence of ZmCYP71Z18 as a highly promiscuous P450 capable of functionally decorating each of the respective *ent*-iso-kaurene, dolabradiene, and 5-rosanol intermediates creates a modular pathway network for the efficient production of bioactive diterpenoid blends ^16,20^. The absence of significant co-regulation patterns (Figs. 2 & 7) and the low product abundance when pairing ZmTPS42/KSL1 with the 8,13-CPP synthase, ZmTPS40/CPS4, or the (+)−CPP synthase, ZmTPS39/CPS3 ^27^ (Fig. 3), support *ent*-CPP as the dominant ZmTPS42/KSL1 substrate. Absence of rosalexins in maize lines with non-functional ZmTPS42/KSL1 genes, highly pathogen-inducible gene expression of ZmTPS42/KSL1, and mGWAS studies revealing the association of rosalexins with the *ZmTPS42/KSL1* genome locus (Figs. 4 & 5), suggest that *ZmTPS42/KSL1* is the primary genetic determinant controlling the biosynthesis of both 5-rosanol and epoxyrosanol and support its central role in rosalexin biosynthesis in maize.

The formation of 5-rosanol by ZmTPS42/KSL1 reveals a previously unknown catalytic mechanism within the functional diversity of the class I diTPS family in plants. Based on known class I diTPS reactions and AI-assisted mechanistic studies we here propose that 5-rosanol formation proceeds by initial biosynthesis of a pimara-15-en-8-yl^+^ carbocation, followed by a series of 1,2-hydride and methyl migrations to yield the final rosa-15-en-5-yl^+^ intermediate, which is neutralized via water quenching (Fig. 3). As such, ZmTPS42/KSL1 expands the small group of known class I diTPSs that add water during catalysis, with other known examples in plants including the *Salvia sclarea* sclareol synthase ^44,45^, *Isodon rubescens* nezukol synthase ^46^, 9-hydroxy-pimarane synthases in switchgrass ^10^, 16-hydroxy-kaurane synthases in *Physcomitrium patens* ^47^, *T. wilfordii* ^48^ and *Populus trichocarpa* ^49^, and 13-hydroxy-8(14)-abietene synthases in several gymnosperm species ^50^. Notably, small amounts of a predicted rosadiene scaffold suggest that the more prototypical termination of the intermediary carbocation via deprotonation does occur during ZmTPS42/KSL1 catalysis (Fig. 3 & 4). Moreover, considering that additional C-19 methyl migration from the rosa-15-en-5-yl^+^ intermediate would lead to the formation of a dolabrane scaffold ^39^, supports a related evolutionary path of ZmTPS42/KSL1 and ZmTPS45/KSL4 consistent with their close phylogenetic relationship (Fig. 2). To current knowledge, the biosynthesis of 5-rosanol is unique to maize, although rosane-type diterpenoids have been identified in a range of plant lineages, for example, liverworts as well as Araucariaceae, Linaceae, and Euphorbiaceae species ^51-54^.

Downstream of 5-rosanol and epoxyrosanol biosynthesis, the occurrence of THR as a major metabolite *in planta* (Fig. 5) - formed via enzymatic or spontaneous opening of the epoxide ring in epoxyrosanol - bears striking resemblance to the production of THD in dolabralexin metabolism ^17^. As similarly observed for dolabralexins ^17^, epoxyrosanol - but to lesser extent 5-rosanol or the THR - show potent antibiotic activity against a range of maize pathogens such as *F. verticillioides*, *A. parasiticus*, and *R. microsporus* tested here (Fig. 6), illustrating that the epoxide functional group is critical for bioactivity in both dolabralexins and rosalexins. Notably, this finding aligns with the often high reactivity and antimicrobial potential of chemoenzymatically generated terpenoid epoxides ^55^. In maize, the functional relevance of these trihydroxy derivatives of epoxydolabrene and epoxyrosanol remains to be uncovered, given their limited antimicrobial activity but substantial abundance *in planta*. Despite the pathogen-elicited accumulation of rosalexins in stem tissue and the potent antimicrobial activity of epoxyrosanol *in vitro* (Figs. 5-7), the absence of a distinctive resistance phenotype in rosalexin-producing *vs* non-functional lines upon *F. verticillioides* infection suggests that rosalexins play a distinct context-dependent or lesser role in the dynamic network of maize chemical defense layers as compared to kauralexins and zealexins ^6^. For example, consistent *ZmTPS42/KSL1* transcript accumulation patterns in aerial tissues have been observed in response to infection by other foliar pathogens such as *Cochliobolus heterostrophus* and *Colletotrichum graminicola* ^16,56^.

The discovery of the rosalexin pathway completes a triad of bioactive diterpenoid groups that evolved from ancestral GA metabolism in maize and predictably contribute to above- (kauralexins, rosalexins) and below-ground (dolabralexins) chemical defenses and other environmental interactions. Characterizing these enzymes and their roles in the modular maize diterpenoid metabolic network provides a framework for understanding the extensive variation in diterpenoid abundance, composition, and stress inducibility across genetically diverse maize lines and commercial hybrids, and how this variation contributes to stress resilience ^17,23^. Ultimately, identifying maize lines with diterpenoid profiles promoting stress resilience provide resources for optimizing resistance traits.

## Methods

### Plant cultivation

Maize seeds were obtained from the USDA National Plant Germplasm System (https://www.grin-global.org/) and the Maize Genetic COOP Stock Center (https://maizecoopsc.org/). For *in planta* detection of rosalexins (Fig. 5), plants were greenhouse grown at the UC San Diego Biology Field Station as previously described ^57^. In brief, maize seeds were sterilized using 70% EtOH followed by a 15% bleach solution before being washed with MilliQ water. Seeds were then germinated in MetroMix 200 (Sun Gro Horticulture Distribution) soil supplemented with 14-14-14 Osmocote (Scotts Miracle-Gro) and plants were cultivated with a 12-h photoperiod with a minimum of 300 μmol m^−2^s^−1^ of photosynthetically active radiation supplied by supplemental lighting, 70% relative humidity, and a day/night temperature cycle of 28/24°C. For rosalexin pathway profiling of selected maize lines (Fig. 7), plants were greenhouse grown at the UC Davis as described above with a few modifications. Here, seeds were germinated in an autoclaved soil mixture of half BM7 35% Bark HP and half Turface Athletics MVP and supplemented with nutrient water (Ca(NO_3_)_2_, KNO_3_, Fe-EDTA, Zn-EDTA, Cu-EDTA, Na_2_MoO_4_, KH_2_PO_4_, MgSO_4_, and K_2_SO_4_). For enzyme functional assays, *N. benthamiana* was grown from seed in Sunshine Mix (SUN GRO) and supplemented with nutrient water (Ca(NO_3_)_2_, KNO_3_, Fe-EDTA, Zn-EDTA, Cu-EDTA, Na_2_MoO_4_, KH_2_PO_4_, MgSO_4_, and K_2_SO_4_) in controlled environment growth chambers under a photoperiod of 12/12 h, 60% relative humidity, 100 μM m^−2^s^−1^ light intensity, and a day/night temperature cycle of 26/25°C.

### Fungal pathogen infection of maize tissues

For stem inoculations with *F. verticillioides* (isolate GL1093), 8-11 cm longitudinal incisions were made between nodes 3 and 4 using a surgical scalpel and treated with 1 mL of fungal spore solution (1×10^7^ mL^−1^) in MilliQ water or with water only as a control. For stem lesion imaging, a BD PrecisionGlide Needle (18G) was used to create a 1.2 mm diameter hole under node 3 and inoculated with 1 mL of fungal spore solution (1×10^7^ mL^−1^) in MilliQ water. Wound sites were wrapped with clear tape to prevent desiccation. For root elicitation assays, soil was gently brushed from nodal roots, which were punctured at 1 cm intervals using a needle and inoculated with 50 μL fungal spore solution (1×10^7^ mL^−1^) in MilliQ water or water only (control) at each wound site. Plants were additionally watered with 600 mL of fungal spore solution (1×10^7^ mL^−1^) in MilliQ water or water (control) to increase root infection. Plants (n=3-8 per genotype) were harvested 7-9 days post infection over the course of 3 days in a random order to account for variation that may have occurred over the collection period. Representative root sections were collected and washed gently with deionized water. All harvested tissue samples were immediately frozen in liquid N_2_ and stored at −80°C for further analysis. Lesion images were taken immediately after harvest.

### Transcriptome sequencing and analysis

For transcriptome analysis, root and stem tissue (n=3) from control and fungal infected plants of maize genotypes selected for the pathogen elicitation assays was used. RNA was extracted using the Quick-RNA Plant Miniprep kit (Zymo Research). Gene expression analysis of the RNA-seq data was performed by Novogene. In brief, RNA sequencing libraries were prepared and sequenced using the Illumina NovaSeq X-Plus Sequencing platform with a 2x150 paired-end read configuration. Raw sequencing data were generated as FASTQ files. A total of 84 libraries were sequenced, yielding raw read counts ranging from 39,892,816 to 76,617,254 reads per sample. The obtained data were processed to remove low-quality sequences and adapter contamination using the Novogene QC pipeline. After filtering, the number of retained reads ranged from 38,794,843 to 67,827,953 reads per sample. Filtered reads from each library were aligned to the maize reference genome (Zm-B73-REFERENCE-NAM-5.0) using HISAT2 version 2.2.1 ^58^. FeatureCounts (2.06) ^59^ was used to count the number of reads mapped to each gene. The resulting averaged Fragments per Kilobase of transcript Million (FPKM) mapped reads were used to calculate the gene expression heat map in RStudio with the ComplexHeatmap package using $\log\;10(\frac{sample\;mean\;[FPKM+1]}{median\;across\;all\;samples\;(FPKM)})$ for the calculations. Transcript abundance fold change values for ZmTPS42/KSL1 between pathogen-infected and control samples were calculated after normalization to the maize housekeeping gene, *ZmEF1-ɑ* (Zm00001eb346230).

### *In vivo* transient co-expression assays in *Nicotiana benthamiana*

Full-length, codon optimized genes of the focal diTPSs and P450s were obtained by DNA synthesis (Twist Bioscience) with financial support by the DOE Joint Genome Institute (JGI) DNA Synthesis Science Program (grant no. 510448) and inserted into the binary pEAQ-HT vector system ^60^. For co-expression in *N. benthamiana*, constructs were transformed into *A. tumefaciens* strain GV3101 and cells were grown at 28°C for 48 hours in LB media supplemented with 10 μL mL^−1^ of gentamicin and rifampicin and 50 μL mL^−1^ of kanamycin. Cells were then adjusted to a final optical density (OD_600_) of 0.5 in 10 mM MES buffer (pH 5.6) with 10 mM MgCl_2_ and 150 μM acetosyringone (Acros Organics). For co-expression of the chosen diTPS and P450 combinations the respective Agrobacterium cultures were combined in equal volumes including the silencing suppressor strain p19 ^61^ and infiltrated into the abaxial side of the leaves of 5-week-old *N. benthamiana* plants using a needless syringe. Five days post-infiltration, metabolite extraction from transfected plants was performed via a 15-minute sonication using 5 mL 85:15 (v/v) hexane:ethyl acetate (Fisher Scientific) and analyzed via GC-MS or LC-MS/MS as described below.

### GC-MS analysis of enzyme products

GC-MS analysis of enzyme products was conducted on an Agilent 7890B GC interfaced with a 5977 Extractor XL MS Detector at 70 eV and 1.2 mL min^−1^ He flow, using an Agilent DB-XLB column (30 m, 250 μm i.d., 0.25 μm film) and the following GC parameters: 50°C for 3 min, 15°C min^−1^ to 300°C, hold for 3 min with pulsed splitless injection at 250°C. MS data from 40–400 mass-to-charge ratio (*m*/*z*) were collected after a 13-min solvent delay. Metabolite quantification (n=4) was based on normalization to the internal standard 1-eicosene (Sigma-Aldrich), tissue dry weight, and OD_600_ at the time of induction of protein expression.

### Metabolite profiling of maize tissues via LC-MS/MS

To quantify plant metabolites, 100 mg of maize tissue was collected from the fungal elicitation experiment (see above). Fresh tissue was lyophilized for 48 h and then ground into a fine powder using a ball Retsch mill. Metabolites were extracted using 80% Optima LC-MS grade MeOH (Fisher Scientific) containing 0.5 μM Telmisartan (Sigma-Aldrich) as internal standard for quantification. Liquid chromatography was performed on a Thermo Scientific Vanquish UHPLC with an Acquity UPLC BEH C18 column (2.1 × 100 mm, 1.7 μm particle size; Waters) using Optima LC-MS grade water with 0.1% formic acid (v/v) as eluent A and Optima acetonitrile with 0.1% formic acid (v/v) as eluent B. The chromatography method used a flow rate of 0.6 mL min^−1^: −1 to 0 min, 1% B, 0 to 0.5 min, 50% B, 0.5 to 7.5 min, 90% B, 7.5 to 9 min, 1% B, 9 to 9 min 1% B, 9 to 10 min 1% B with the column compartment set to 40°C. Mass spectrometry was performed using a Thermo Scientific Q-Exactive equipped with an electrospray ionization source that was run in positive ionization mode (spray voltage, 3.50 ∣kV∣; capillary temperature, 300°C; aux. gas heater, 350°C; sheath gas flow rate, 45; aux. gas flow rate, 10; sweep gas flow rate, 3). The MSDial software program ^62^ was used to generate and analyze the LC-MS/MS generated data. To quantify metabolites, peak area was autointegrated and extracted from Thermo Scientific Free Style and all calculations were performed in Excel (**Supplementary Table S5**).

### *In vitro* fungal growth inhibition assays

Fungal growth inhibition assays were performed following the Clinical and Laboratory Standards Institute M38-A2 guidelines as described previously ^19^ using *F. verticillioides* GL1093 (kindly provided by Dr. Eric Schmelz), *A. parasiticus* (NRRL #6433), and *R. microsporus* (NRRL #5558). Fungal isolates were cultured on potato dextrose agar for three days at room temperature before the quantification and use of spores ^63,64^. Spore densities were estimated using a hemocytometer and initial fungal inoculum was diluted with RPMI-1640 MOPS buffer (pH 7) to 2.5 × 10^4^ conidia mL^−1^. Fungal growth at 30°C was monitored using a Infinite M Nano microplate absorbance reader (Tecan) with a 96-well microtiter plate-based method. Each well contained 200 μL of initial fungal inoculum with 2.5 μL of pure DMSO or RXs in DMSO at concentrations of 10, 25, and 50 μg mL^−1^. Negative and positive controls consisted of 200 μL of RPMI-1640 MOPS or initial fungal inoculum, respectively, each containing 2.5 μL of pure DMSO. Averaged changes in OD_600_ (n=6) were calculated hourly over 48 hours by measuring OD_600_ values normalized to the time point 0 OD_600_ and then to the OD_600_ of the negative control at each corresponding time point. Data were assessed via Analysis of Variance (ANOVA) followed by a Tukey Honest Significant Difference (HSD) statistical analysis. Area under the curve (AUC), approximated under the Trapezoidal Rule, was used to calculate fold change in growth for the statistically significant concentrations relative to the positive control. Uncertainty was estimated by Monte Carlo simulations [CI 95%, n = 5000] to propagate replicate variation into AUC comparisons.

### Chemical synthesis and purification of trihydroxyrosanol from epoxyrosanol

Trihydroxyrosanol (THR) was generated by acid-catalyzed epoxide opening of epoxyrosanol. Purified epoxyrosanol (2 mg) was dissolved in 4 mL of 60% (v/v) acetone and vortexed thoroughly, followed by adding 220 μL of 0.5 M H_2_SO_4_ and incubating at room temperature for 12 h. The reaction was quenched by the dropwise addition of concentrated NaHCO_3_ until effervescence ceased, followed by the addition of 4 mL saturated NaCl brine. The product was extracted thrice with 1 mL of 9:1 (v/v) ethyl acetate:methanol. Combined organic layers were washed with 600 μL brine, dried over anhydrous Na_2_SO_4_, and transferred to a clean vial. Conversion and purity were verified by LC-MS/MS. Purification was performed using a semi-preparative Agilent 1100 Series HPLC system equipped with an Agilent Eclipse XDB-C18 column (5 μm, 4.6 × 150 mm). A gradient elution was performed with solvent A (Milli-Q water) and solvent B (HPLC-grade acetonitrile) at a flow rate of 1 mL min_−1_. The gradient profile was 0 min 20% B to 30 min 40% B. The purified THR fraction was collected and analyzed by NMR in MeOH-d_4_ as described below.

### Experimental NMR analysis

For NMR analysis, ≥1 mg of diterpenoid products was enzymatically produced via transient expression in *N. benthamiana* as outlined above. The reaction products were extracted in 85:15 (v/v) hexane:ethyl acetate and purified via separation on a 500 mL silica matrix with a hexane/ethyl acetate gradient as the mobile phase and subsequent semi-preparative chromatography on Agilent 1100 Series HPLC equipped with an Agilent Eclipse XDB-C18 5 μm 4.6x150 mm column using a gradient elution with eluent A 100% MilliQ water and eluent B HPLC-grade acetonitrile. Products were eluted under the following conditions: Epoxyrosanol: 0 min 60% B to 30 min 90% B; 5-rosanol: 0 min 80% B to 30 min 100% B; epoxyrosaene: 0 min 50% B to 30 min 100% B with 1 mL fractions collected over 30 min at a flow rate of 1 mL min^−1^. Purified products were dissolved in deuterated chloroform-d (CDCl_3_; Sigma-Aldrich) containing 0.03% (v/v) tetramethylsilane (TMS). The 1D (1H, 13C, NOE) and 2D (HSQC, COSY, HMBC, H2BC and NOESY) spectra were acquired on a Bruker Avance III 800 MHz spectrometer equipped with a 1H-13C / 15N / 2H Bruker CPTCI Cryoprobe. NMR spectra were analyzed using Bruker TOPSPIN 3.2.

### Computational stereochemical analysis of 5-rosanol

The target diastereomer was predicted by comparing experimental proton and carbon NMR chemical shift values to calculated values for the 16 possible structures ^65^. For each diastereomer, the method begins with a conformational search. Low energy conformations were found using xTB-CREST, then optimized using Gaussian 16 with a restricted B3LYP-D3(0)/6-31+G(d,p) level of theory and implicit solvation model (IEFPCM) for chloroform ^66-71^. Conformations within 3 kcal/mol of the lowest energy conformers were used to calculate computational NMR shifts using the GIAO method at the mPW1PW91/6-311+G(2d,p) level, and chloroform as the solvent ^72,73^.

Both proton and carbon isotropic shielding values were computed. These computed isotropic shielding values were converted using CHESHIRE to predicted NMR shifts which were weighted based on their calculated free energy in the optimization calculation and averaged using the referenced code ^65^. The predicted NMR shifts for each of the diastereomers were compared to the experimental NMR shifts using DP4+ to determine which diastereomer’s predicted shifts were most similar to the experimental shifts ^74^. This method resulted in isomer 11 having the closest predicted shifts to the experimental with a computed probability of 100%.

### Computational analysis of the *Zm*TPS42/KSL1 reaction mechanism

The free energy profile for the formation of 5-rosanol was generated using RxnNet ^33^, an AI-assisted automated reaction mechanism discovery platform. RxnNet requires only the SMILES corresponding to the initial and final carbocations as the input. It automatically identifies the reaction cascade between the reactant and product and constructs the corresponding free energy diagram. RxnNet generates the minimum energy path (MEP) for each step of the reaction in a stepwise manner, and generates near-attack conformers (NAC) ^34,35^ for intermediates prior to performing the actual chemical step. For all chemical and conformational steps, an initial MEP guess is generated with the help of geodesic interpolation ^75^, followed by refinements using climbing image nudged elastic band (NEB-CI) calculations. A NEB-CI is initially performed at the semi-empirical quantum chemistry (QC) level of theory using GFN2-XTB ^34^. The final MEP is then obtained from NEB-CI using M06-2X/def2-SVP level of theory ^76-78^, and based on this MEP RxnNet identifies stationary points. RxnNet employs ORCA 6.0.1 ^79,80^ to perform all QC calculations. Readers can refer to reference ^33^ for further details on RxnNet.

### Mutual rank and Pearson correlation coefficient calculation of genes

RNA-seq datasets of infected and control (n=3) stem tissue from maize lines Oh7B, Ki3, CML103, and CML277 were used for analyzing Mutual Rank (MR) and Pearson correlation coefficients (PCC). Raw FPKM values were log2[FPKM+0.001] transformed and zero variance genes were removed prior to analysis. *ZmTPS38/CPS2/AN2* and *ZmTPS42/KSL1* were used as bait genes and tested against *kauralexin reductase 2* (*ZmKR2*) as well as TPS and CYP genes involved in specialized metabolism and GA biosynthesis. PCC values were calculated using pairwise complete observations. To assess correlation strength and specificity, a MR analysis was performed for each gene pair. PCC values were generated for each gene in the gene pair against all expressed genes in the dataset, and the MR value was calculated from the geometric mean between these two ranks. Gene pair strength was visualized using heat maps.

### Sequence and Phylogenetic Analysis

Sequence alignments of diTPS genes were generated using the CLCBio Workbench (Qiagen) with default parameters. A maximum-likelihood phylogenetic tree was generated using PhyML ^81^ with four rate substitution categories, LG substitution model, BIONJ starting tree, and 500 bootstrap repetitions.

### Metabolite-based Genome Wide Association Studies (mGWAS)

The Goodman diversity panel was planted in summer 2016 at the UC San Diego Biology Field Station. To minimize variation in plant responses arising from differential recognition or activity of fungal effectors across diverse maize lines, all mapping experiments used heat-killed *F. venenatum* hyphae combined with slit-stem elicitation. Maize stems were harvested 5 days after elicitation, immediately frozen in liquid nitrogen, ground into a fine powder, and stored at −80°C for subsequent analyses. Metabolite-based Genome-wide association studies (mGWAS) were conducted against 5-rosanol (*m*/*z* 275) and epoxyrosanol (*m*/*z* 291) using the imputed resequencing SNP panel, aligned to the B73 RefGen_V5 maize reference genome, obtained from Dryad ^82^. Using TASSEL (v5.2.96) ^83^, chromosome files were merged, restricted to biallelic SNPs, subset to the 228 phenotyped inbreds, converted to homozygous calls, and filtered for a site minimum count of 20 and allele frequencies between 0.05 and 0.95, yielding 12,343,358 SNPs. Metabolite abundances were transformed as log_2_(x+1). Mixed linear model (MLM) association analysis was run in rMVP (v1.0.8) with a VanRaden kinship matrix and the first five principal components as covariates (EMMA) ^84^. Manhattan plots were generated using the Python package Assocplots ^85^.

## Supplementary Material

**Supplementary Fig. S1:** Protein sequence alignment of ZmTPS42/KSL1 derived from selected maize lines.

**Supplementary Fig. S2:** Mass spectra of products from the pairwise reaction of ZTPS42/KSL1 with different maize class II diterpene synthases.

**Supplementary Fig. S3:** NMR structural elucidation of compound **1** (5-rosanol).

**Supplementary Fig. S4:** MEP plots of the chemical steps in the ZmTPS42/KSL1-catalyzed mechanism, as obtained from RxnNet.

**Supplementary Fig. S5:** NMR structural elucidation of compound **13** (epoxyrosanol).

**Supplementary Fig. S6:** NMR structural elucidation of compound **14** (epoxyrosaene).

**Supplementary Fig. S7:** Functional analysis of maize P450 enzymes for 5-rosanol conversion.

**Supplementary Fig. S8:** NMR structural elucidation of compound **15** (trihydroxyrosanol, THR).

**Supplementary Fig. S9:** Recovered epoxyrosanol and trihydroxyrosanol (THR) from fungal bioactivity assays.

**Supplementary Table S1:** FPKM values associated with gene expression analysis of diterpenoid-metabolic pathways.

**Supplementary Table S2:** Stereochemistry of tested computational NMR structures for 5-rosanol

**Supplementary Table S3:** Computational NMR chemical shifts for 5-rosanol.

**Supplementary Table S4:** MS feature area alignment.

**Supplementary Table S5:** LC-MS/MS quantification calculations.

**Supplementary Table S6:** Bonferroni-significant SNPs associated with stem 5-rosanol and epoxyrosanol.

**Supplementary Table S7:** Summary of genes in 5-rosanol and epoxyrosanol GWAS loci, with top MMseqs2 hits to Arabidopsis and rice.

**Supplementary Table S8:** Gene expression in the GWAS mapping region.

**Supplementary Table S9:** Raw reads from Tecan analysis of fungal bioactivity assays

**Supplementary Table S10:** Quantitative metabolite profiling of pathogen-treated maize lines.

Additional supporting information may be found online in the Supplementary Data section at the end of the article.

This is a list of supplementary files associated with this preprint. Click to download.

- Cowieetal.SupplementaryTableS3.xlsx
- Cowieetal.SupplementaryTableS2.xlsx
- Cowieetal.SupplementaryTableS7.xlsx
- Cowieetal.SupplementaryTableS7.xlsx
- Cowieetal.SupplementaryTableS6.xlsx
- Cowieetal.SupplementaryTableS6.xlsx
- Cowieetal.SupplementaryTableS9.xlsx
- Cowieetal.SupplementaryTableS8.xlsx
- Cowieetal.SupplementaryTableS5.xlsx
- CowieSupplementalFiguresCombined.pdf
- Cowieetal.SupplementaryTableS4.xlsx
- Cowieetal.SupplementaryTableS10.xlsx
- Cowieetal.SupplementaryTableS1.xlsx

## Data Availability

The RNA-sequencing data reported in this study were submitted to the Sequence Read Archive (SRA), accession no. PRJNA1454089. Reported NMR data have been deposited at https://www.npmrd-project.org/ with the following IDs: 5-rosanol NP0354244, epoxyrosanol NP0354245, epoxyrosaene NP0354246, and trihydroxyrosanol NP0354247. Computational NMR files have been deposited here: https://iochem-bd.bsc.es/browse/handle/100/492343. Computational energy data have been deposited here: https://iochem-bd.bsc.es/browse/handle/100/492711.

## Significance Statement:

Plant diterpenoids play critical roles in crop development, stress defense and ecological adaptation. In maize, diterpenoids serve as core components of chemical defenses against pests and diseases with direct impact on crop immunity and vigor. Enzymes of the diterpene synthase and cytochrome P450 monooxygenase families largely drive diterpenoid chemical diversity. This study reports the discovery and characterization of the pathway forming rosalexin diterpenoids in maize. Pathogen-elicited accumulation and in vitro antifungal activity of rosalexin metabolites supports a physiological function in maize ecological adaptation.
